# An Autologous Tooth-Shell Technique for Ridge Reconstruction and Immediate Implant Placement: A Two-Case Technical Report and Review of the Literature

**DOI:** 10.3390/jfb17080402

**Published:** 2026-08-14

**Authors:** Jan Łoś, Justyna Szałkowska-Łoś, Michał Pikuła, Paulina Adamska

**Affiliations:** 1 Laboratory of Tissue Engineering and Regenerative Medicine, Division of Embryology, Faculty of Medicine, Medical University of Gdansk, 80-210 Gdansk, Poland; jan.janlos.o@gmail.com (J.Ł.); pikula@gumed.edu.pl (M.P.); 2 Dental Clinic Potokowa, 80-283 Gdansk, Poland; 3 Dental Clinic Happy Smile, 80-369 Gdansk, Poland; 4 Division of Oral Surgery and Implantology, Faculty of Medicine, Medical University of Gdansk, 80-210 Gdansk, Poland

**Keywords:** alveolar ridge augmentation, autografts, dental implant, dentin, dentistry, impacted tooth, oral surgical procedures, third molar

## Abstract

**Background**: Autologous dentin has emerged as a potential grafting material in regenerative oral surgery because of its structural and biological similarities to bone. This technical report describes a modified tooth-shell technique used for horizontal ridge reconstruction with simultaneous implant placement in two clinical cases involving the aesthetic zone. **Case Presentation**: The first case involved a 29-year-old female patient requiring extraction of a partially impacted, buccally positioned maxillary third molar (tooth 28). The extracted tooth was processed intraoperatively to obtain a dentin shell, which was fixed as a buccal plate during simultaneous implant placement. The remaining defect was filled with sticky bone prepared from biphasic calcium phosphate combined with platelet-rich fibrin. Radiographic evaluations performed at 4 and 6 months demonstrated stable ridge contours, while clinical follow-up continued for 2 years with maintenance of implant function and peri-implant tissue stability. The second case involved a 58-year-old female patient with a previous failure of immediate implantation in the anterior maxilla associated with implant overload, loss of implant stability, and buccal bone deficiency. After soft- and hard-tissue healing, ridge reconstruction was performed using a dentin shell prepared from tooth 18 together with simultaneous implant placement and immediate provisional restoration. Follow-up after 6 months revealed stable peri-implant tissues, a satisfactory aesthetic outcome, and successful prosthetic rehabilitation. **Conclusions**: The modified tooth-shell technique using autologous dentin provided satisfactory preliminary clinical and radiographic outcomes in both cases. These findings should be considered hypothesis-generating and require confirmation in larger prospective studies with longer follow-up periods.

## 1. Introduction

Alveolar ridge reconstruction remains a significant clinical challenge in implant dentistry, particularly in patients presenting with a thin buccal bone plate, post-extraction defects, or previous implant failure. Adequate bone volume is essential not only for implant stability and long-term functional success but also for achieving satisfactory aesthetic results, especially in the anterior maxilla [1,2].

A number of augmentation techniques have been introduced to manage deficient ridges, including autogenous block grafts, guided bone regeneration (GBR), xenogeneic grafting materials, and cortical shell procedures, such as the Khoury technique. Although these methods are widely used and supported by clinical evidence, they may be associated with disadvantages, such as donor-site morbidity, extended surgical time, graft resorption, membrane exposure, and postoperative discomfort [3,4,5].

The cortical shell technique described by Fouad Khoury has shown predictable outcomes in horizontal ridge augmentation using thin cortical plates harvested from the retromolar region. Despite its effectiveness, the need for bone harvesting from a second surgical site increases the invasiveness of the procedure and may negatively affect patient comfort and recovery [3,4,6,7].

In recent years, growing attention has been directed toward the use of autologous dentin as a grafting material in regenerative dentistry. Dentin shares many structural and biological features with bone. Both tissues are composed mainly of hydroxyapatite and type I collagen and contain bioactive proteins involved in tissue remodeling and healing, including bone morphogenetic proteins (BMPs), dentin matrix protein-1 (DMP-1), and transforming growth factor beta (TGF-β). Previous experimental and clinical studies suggest that processed dentin may support bone regeneration through osteoconductive and -inductive mechanisms while maintaining favorable volumetric stability during healing [8,9,10,11,12,13,14,15].

Tooth-derived graft materials have already been investigated in procedures such as socket preservation, sinus floor elevation, and horizontal ridge augmentation. However, the use of intact dentin shells as a structural substitute for cortical bone has been described less frequently in the available literature [9,10,13,16].

From a biological perspective, dentin demonstrates properties comparable to those of native bone tissue. Approximately 70% of dentin consists of hydroxyapatite, whereas the organic component is composed mainly of type I collagen and non-collagenous proteins associated with mineralized tissue remodeling. Among the most relevant molecules are BMPs, dentin sialophosphoprotein (DSPP), DMP-1, osteocalcin, osteopontin, and TGF-β. These proteins are believed to influence angiogenesis, osteoblast differentiation, and mineralization during bone healing. Experimental studies have also demonstrated the ability of dentin-derived proteins to stimulate mineral deposition in surrounding tissues [8,9,10,11,12,13,14,15].

Dentin additionally provides favorable mechanical properties. Its rigidity and compressive strength are similar to cortical bone, which allows it to function as a stable shell during ridge reconstruction. Compared with collagen membranes, dentin undergoes slower remodeling and resorption, helping maintain graft volume during the early healing phase. Some authors have also suggested that dentin placed in direct contact with bone may undergo gradual ankylotic integration and replacement remodeling, similar to retained root fragments observed after decoronation procedures [8,9,10,11,12,13,14,15,16,17].

The autologous tooth-shell technique is based on processing an extracted tooth into a thin dentin shell that is fixed to the deficient alveolar ridge and used as a biologic scaffold for regeneration. The space between the shell and recipient bone is typically filled with particulate dentin or additional grafting material to support new bone formation. One of the main advantages of this approach is the use of autologous dentin as a structural barrier, which avoids cortical bone-block harvesting and may potentially reduce donor-site morbidity and postoperative discomfort. Published clinical reports have shown encouraging results in horizontal ridge augmentation, with predictable hard-tissue regeneration and high implant survival rates [18,19,20,21,22].

**Figure 1:**
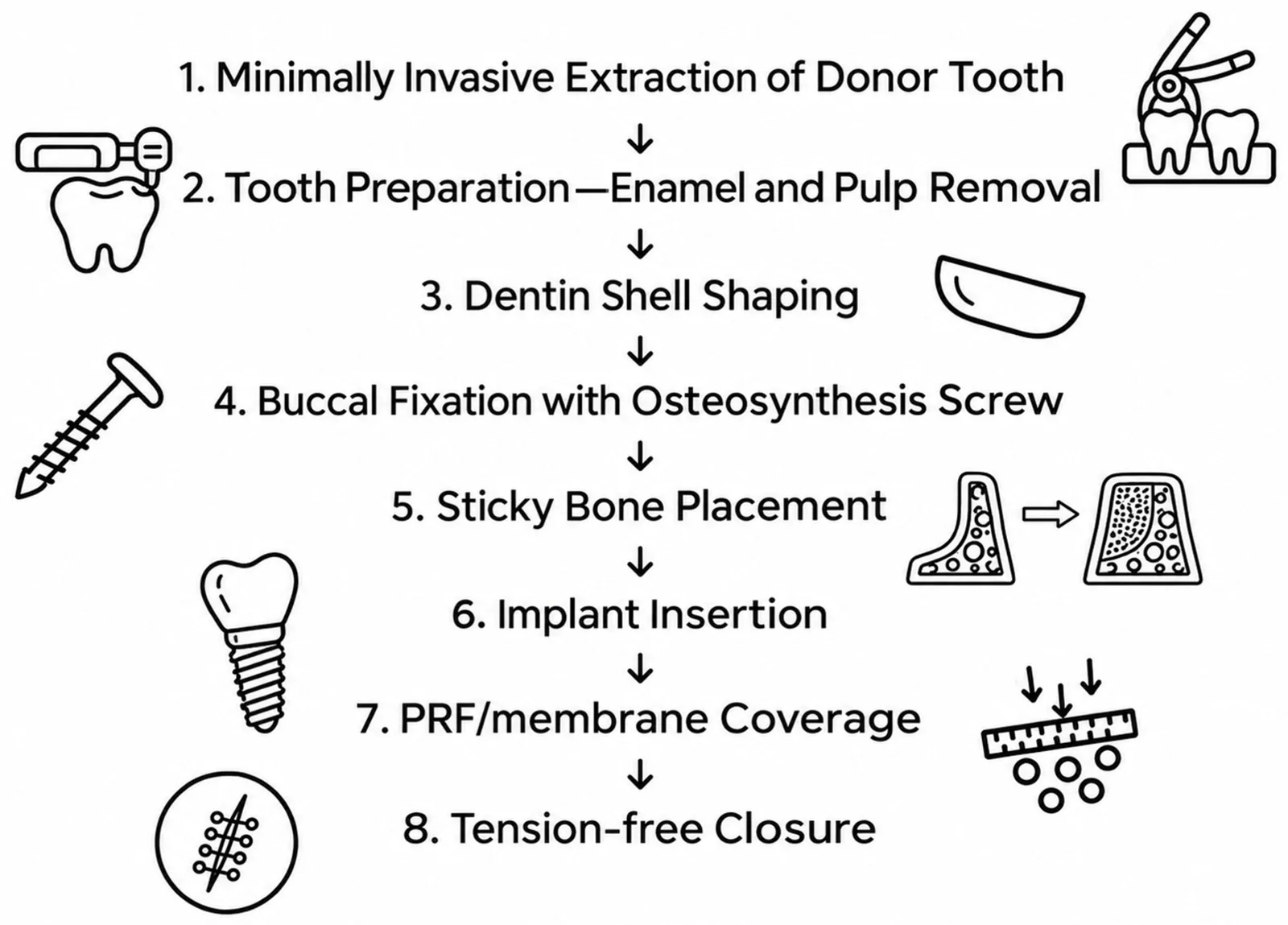
Surgical workflow of modified tooth-shell technique.

This article describes a modified autologous tooth-shell approach combining dentin shell reconstruction, sticky bone augmentation, simultaneous implant placement, and immediate provisionalization within a single surgical protocol. The proposed workflow is presented schematically in Figure 1.

This study presents two clinical cases demonstrating the use of autologous dentin shells for horizontal ridge reconstruction in both primary and salvage immediate implantation scenarios.

## 2. Case Presentation

This technical report introduces a modified autologous tooth-shell technique in which extracted third molars are processed intraoperatively into dentin shells and utilized as buccal structural barriers during simultaneous ridge reconstruction and immediate implant placement. The technique combines principles of autologous dentin grafting, sticky bone augmentation, and cortical shell stabilization while avoiding the need for cortical bone-block harvesting.

### 2.1. Patient 1

#### 2.1.1. Patient 1 Data

A 29-year-old female patient (ASA I) presented for implantological consultation due to the absence of the left maxillary canine. During the medical interview, she reported a history of endometriosis. She was a non-smoker and presented no other systemic risk factors.

**Figure 2:**
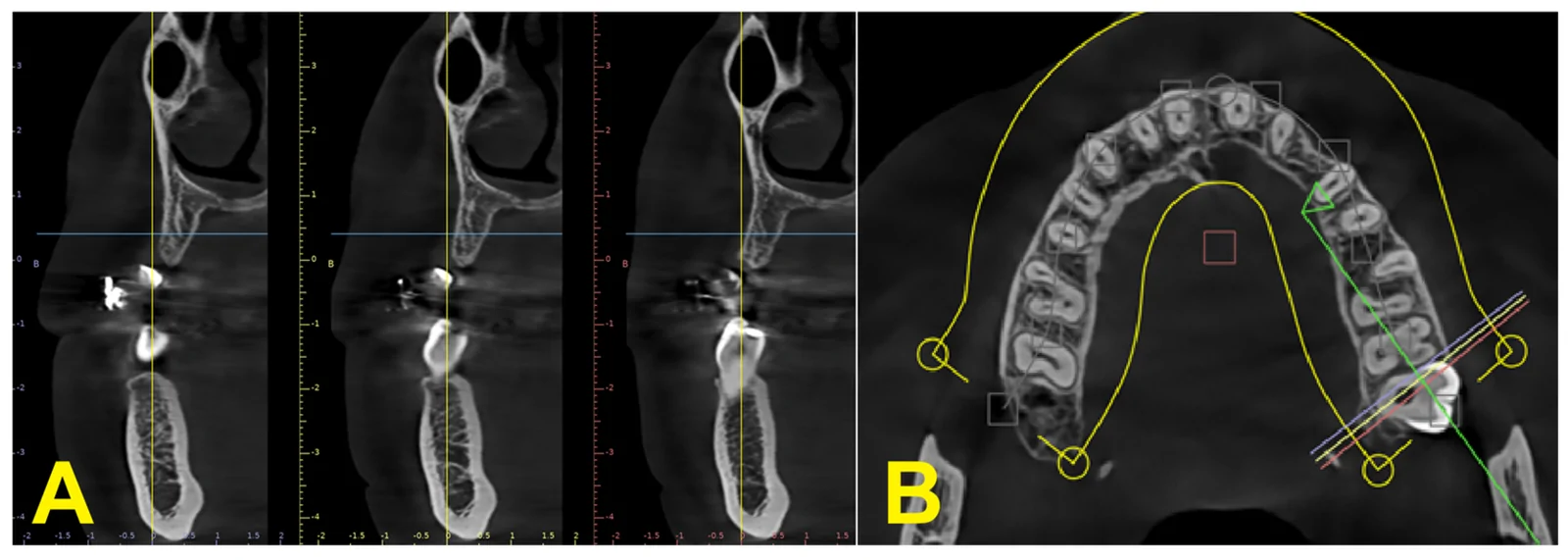
Cone-beam computed tomography of maxillary left canine region before surgery: (**A**) cross-sectional views; (**B**) axial view.

**Figure 3:**
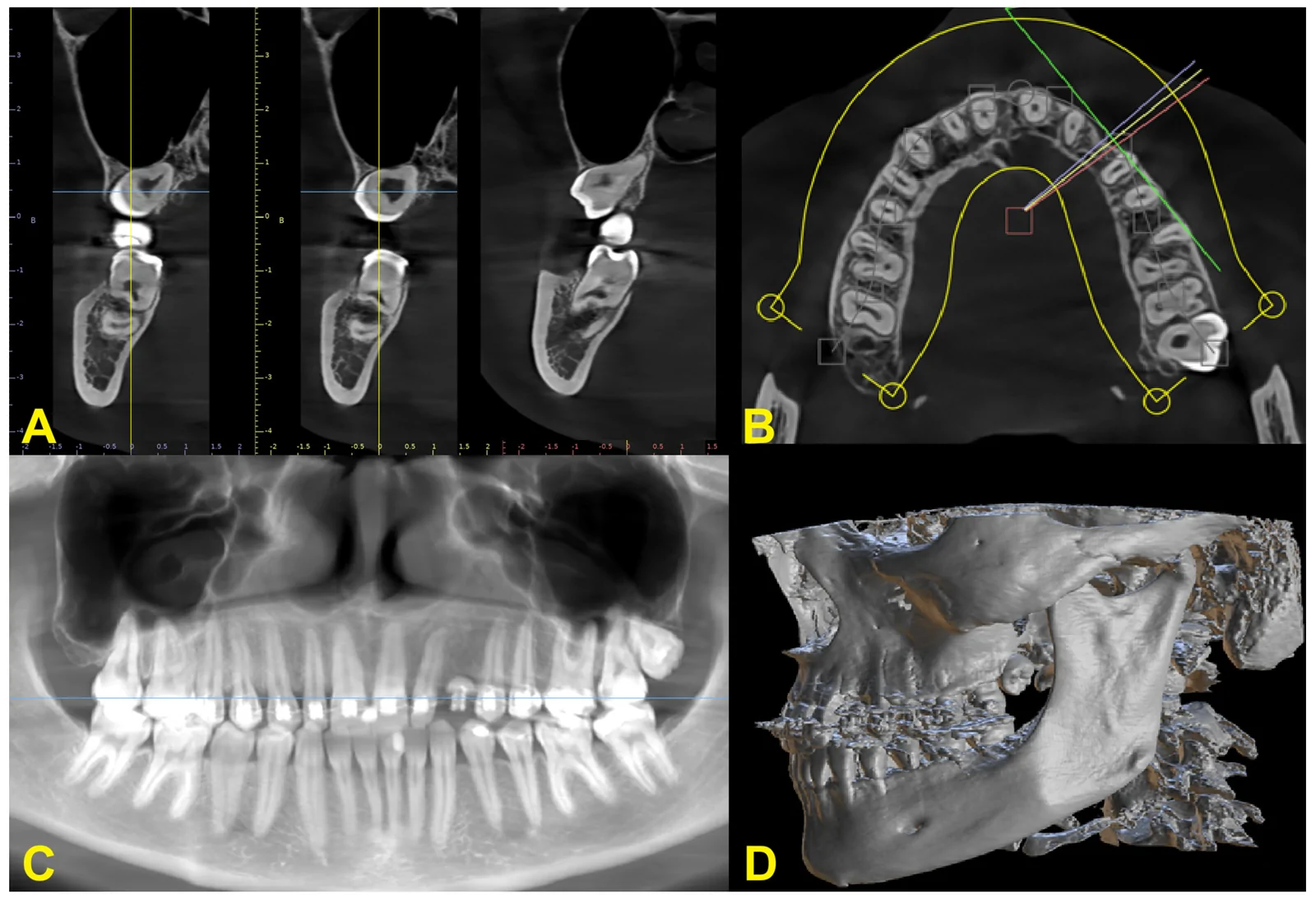
Cone-beam computed tomography of maxillary left third molar: (**A**) cross-sectional views; (**B**) axial view; (**C**) orthopantomographic view; (**D**) pseudo-3D reconstruction.

Clinical and radiographic examination, including cone-beam computed tomography (CBCT) (Figure 2 and Figure 3), revealed the absence of the maxillary left canine (tooth 23) and a partially retained maxillary left third molar (tooth 28, according to the FDI World Dental Federation notation), without signs of localized inflammation.

CBCT imaging revealed a narrow alveolar ridge at the planned implant site, measuring approximately 4.1 mm in width and 14 mm in height. The impacted tooth 28 had partially erupted toward the buccal side. No signs of infection or bone loss were observed in the surrounding area.

Because of its position and close relationship to the outer contour of the alveolar ridge, the retained tooth was considered suitable for modification and use as a natural buccal wall substitute.

#### 2.1.2. Surgical Technique

The surgical procedure was performed under local anesthesia. Nerve conduction was blocked using 4% articaine hydrochloride with 1:100,000 epinephrine (Septodont, Lancaster, PA, USA). A 15C scalpel blade (Swann-Morton, Sheffield, UK) was used, and a mucoperiosteal flap was raised. A 4.0 × 10 mm KISPlant implant (KISPlant, Kuwotech, Gwangju, Republic of Korea) was placed using a 1:20 implant contra-angle (W&H Dentalwerk Bürmoos GmbH, Bürmoos, Austria) at 1200 and 50 rpm, with abundant irrigation using 0.9% NaCl, achieving a primary stability of 45 Ncm. The third molar was extracted using a minimally invasive approach with conventional rotary instruments mounted on a surgical straight handpiece (S-11 LG, W&H Dentalwerk Bürmoos GmbH, Bürmoos, Austria) at 40,000 rpm, with abundant irrigation using 0.9% NaCl.

The tooth was cleaned of all soft tissues, the pulp was removed, and the enamel was completely stripped, leaving only mineralized dentin. The dentin fragment was reshaped using a diamond bur and adapted to the buccal contour of the alveolus. The prepared dentin shell measured approximately 2 mm in thickness, 8 mm in height, and 9 mm in width, and it was stabilized with a single titanium osteosynthesis screw measuring 1.4 × 6 mm (Integra, Warszawa, Poland), securing it to the alveolar crest to serve as a rigid buccal wall. Sticky bone was prepared intraoperatively using approximately 0.8–1.0 mL of R.T.R.+ 40/60 biphasic calcium phosphate granules (Septodont, Saint-Maur-des-Fossés, France), composed of 60% hydroxyapatite and 40% tricalcium phosphate, with a particle size of 0.5–1.0 mm. The granules were mixed with autologous advanced platelet-rich fibrin (A-PRF) and injectable platelet-rich fibrin (iPRF) to enhance graft cohesiveness and handling. Before surgery, four 10 mL tubes of venous blood were collected into sterile, anticoagulant-free, glass-coated plastic tubes and immediately centrifuged using an All Centrifuge device (Scilogex, LLC, Rocky Hill, CT, USA). The blood was centrifuged at 1500 rpm for 14 min to obtain A-PRF and at 700 rpm for 3 min to obtain iPRF. The A-PRF clots were then placed in a PRF box (Quadrostom, Kraków, Poland). The graft mixture was placed between the buccal dentin wall and inner socket. A resorbable bilayer PLGA membrane (R.T.R.+ Membrane, Septodont, Saint-Maur-des-Fossés, France) was placed over the augmented area to support graft containment and soft-tissue healing. Primary closure was achieved without tension.

**Figure 4:**
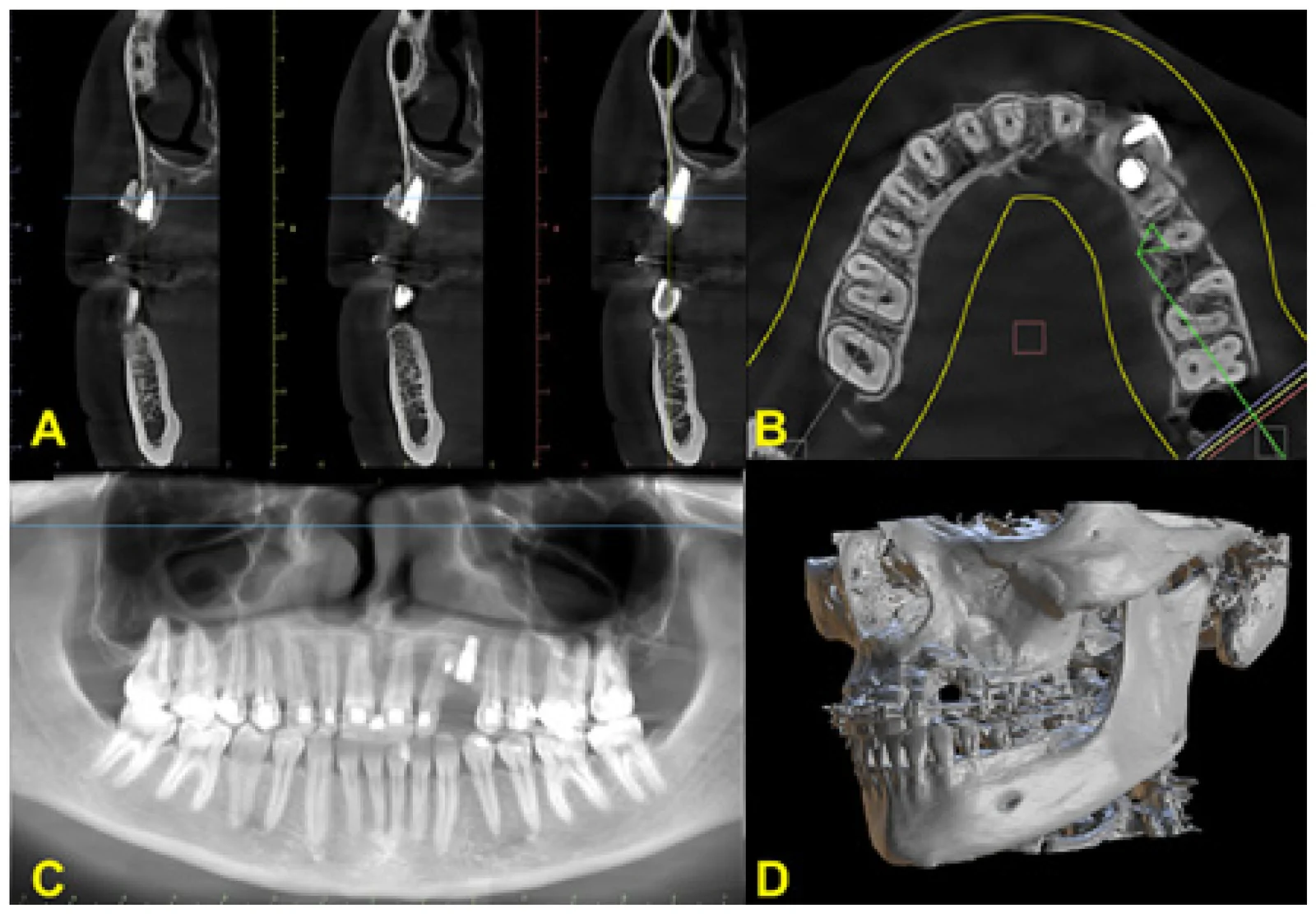
Cone-beam computed tomography of maxillary left canine region immediately after surgery: (**A**) cross-sectional views; (**B**) axial view; (**C**) panoramic reconstruction; (**D**) pseudo-3D reconstruction.

Immediate postoperative CBCT imaging confirmed proper positioning of the implant and dentin shell (Figure 4).

#### 2.1.3. Postoperative Care

Standard antibiotic prophylaxis (amoxicillin at 2 g/day for 5 days [0.875 g + 0.125 g; Augmentin, GlaxoSmithKline, London, UK]) and chlorhexidine rinses (0.12%; Eludril Classic, Pierre Fabre Oral Care, Lavaur, France) were prescribed. Sutures were removed after 10 days. Healing was generally uneventful. A minor soft-tissue dehiscence occurred around the osteosynthesis screw, resulting in a small mucosal scar without graft exposure, infection, or the need for additional surgical intervention.

#### 2.1.4. Prosthetic Reconstruction

**Figure 5:**
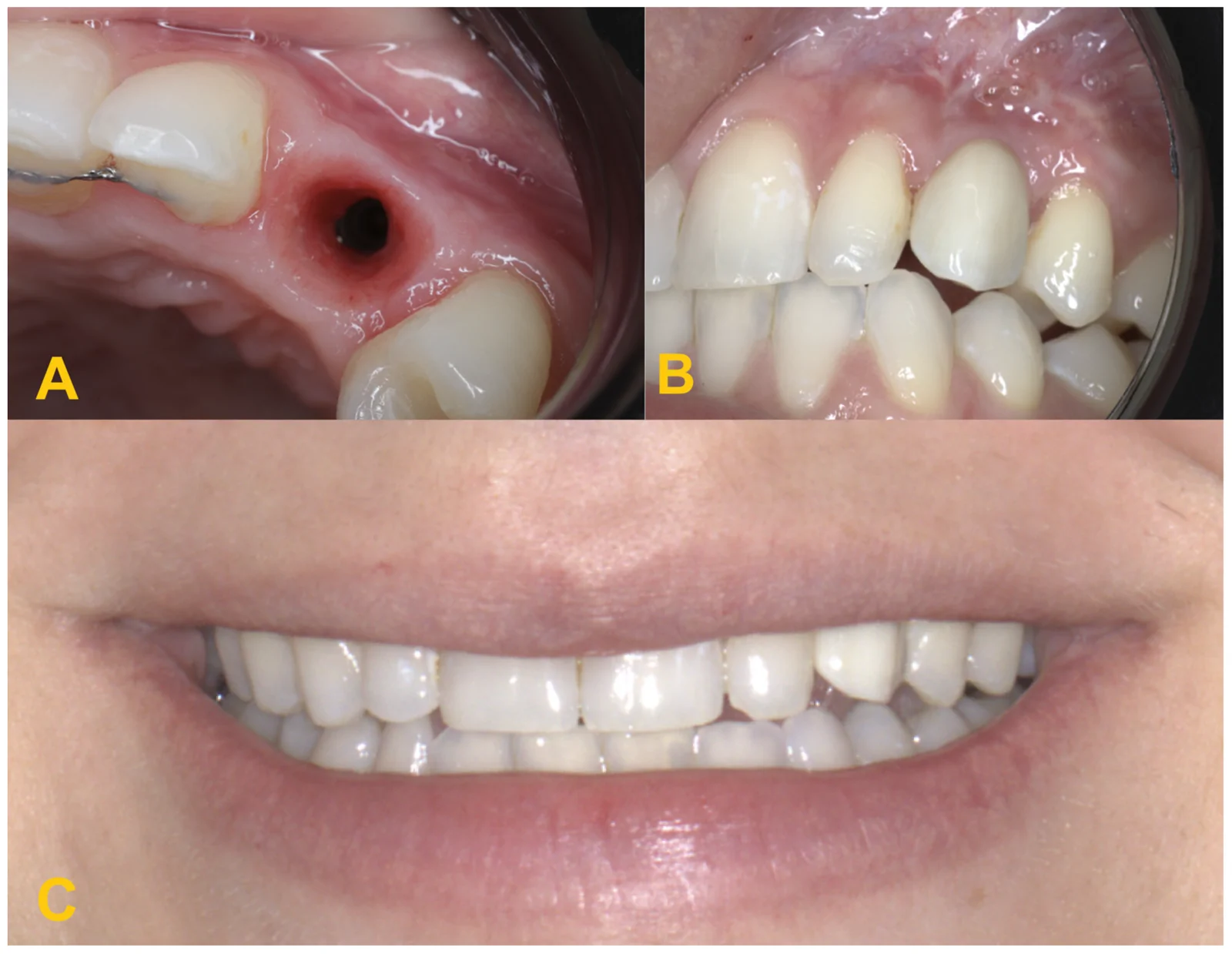
Clinical outcome and definitive prosthetic restoration in Case 1: (**A**) peri-implant emergence profile; (**B**) definitive crown after placement in dental arch; (**C**) full-smile view.

**Figure 6:**
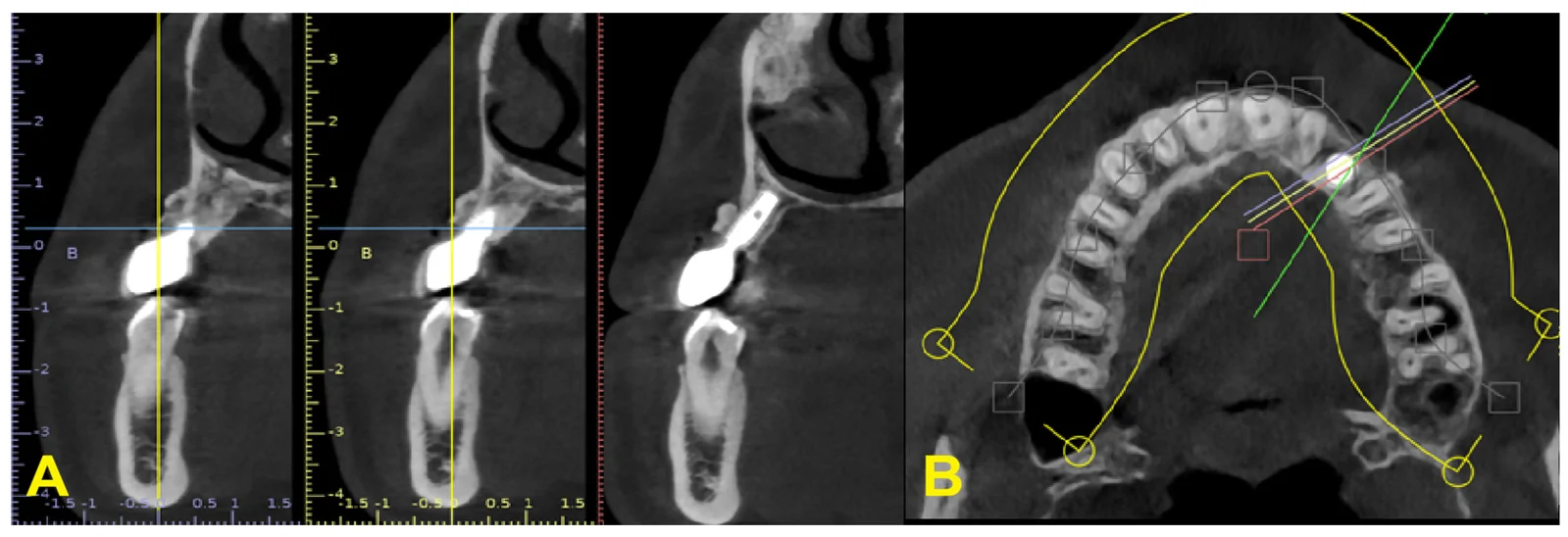
Cone-beam computed tomography of maxillary left canine position after prosthetic crown delivery: (**A**) cross-sectional views; (**B**) axial view.

The implant was subsequently restored with a definitive zirconia-based porcelain crown. CBCT examinations performed at 4 and 6 months demonstrated stable ridge dimensions and satisfactory peri-implant bone contours. Clinical follow-up was continued for 2 years, during which the implant remained stable and functional, with preserved hard-tissue dimensions, healthy peri-implant soft tissues, and a satisfactory aesthetic outcome (Figure 5 and Figure 6).

### 2.2. Patient 2

#### 2.2.1. Patient 2 Data

A 58-year-old female patient with controlled hypertension (ASA II) came in for implantology consultation regarding immediate implantation in the region of tooth 21 (maxillary left first incisor). The patient was a non-smoker and reported no additional systemic diseases.

**Figure 7:**
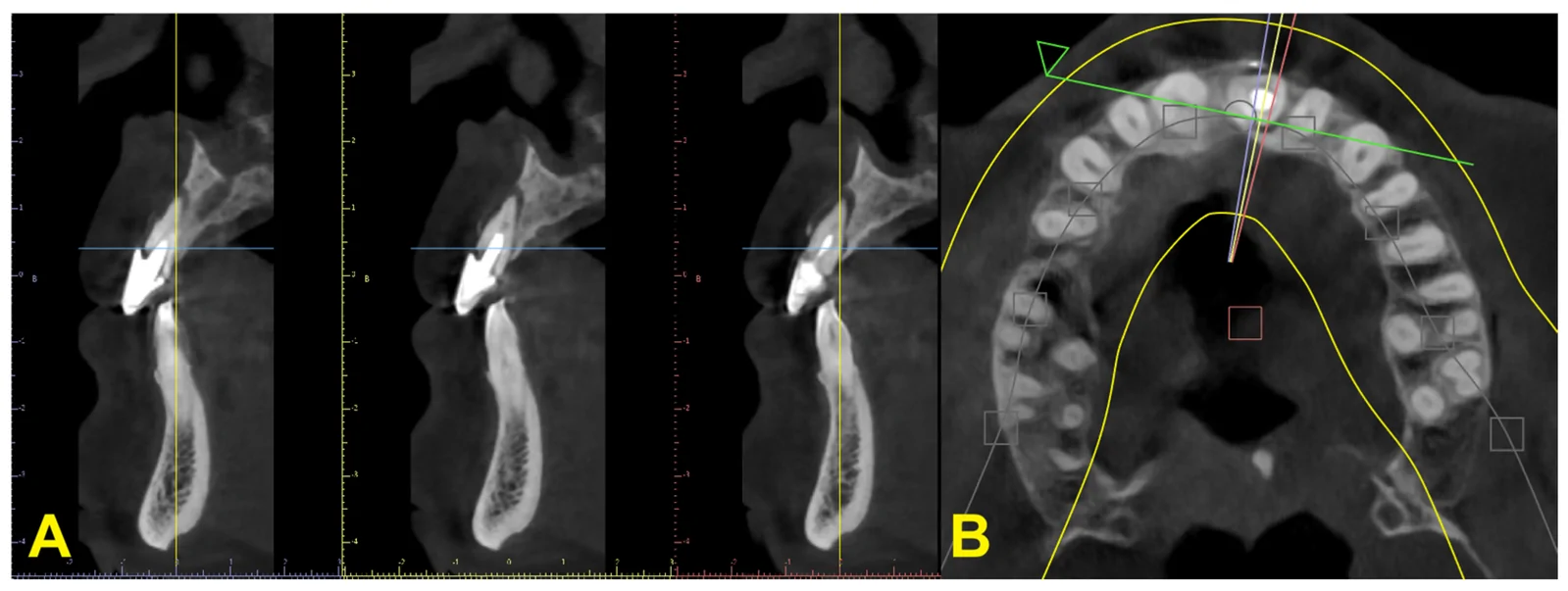
Cone-beam computed tomography of tooth 21 before extraction: (**A**) cross-sectional views; (**B**) axial view.

Clinical and radiographic evaluation (Figure 7) revealed a vertical root fracture associated with a previously restored tooth supported by a post-and-core reconstruction. Because of the unfavorable prognosis, extraction with immediate implantation was planned.

#### 2.2.2. Initial Implant Procedure

**Figure 8:**
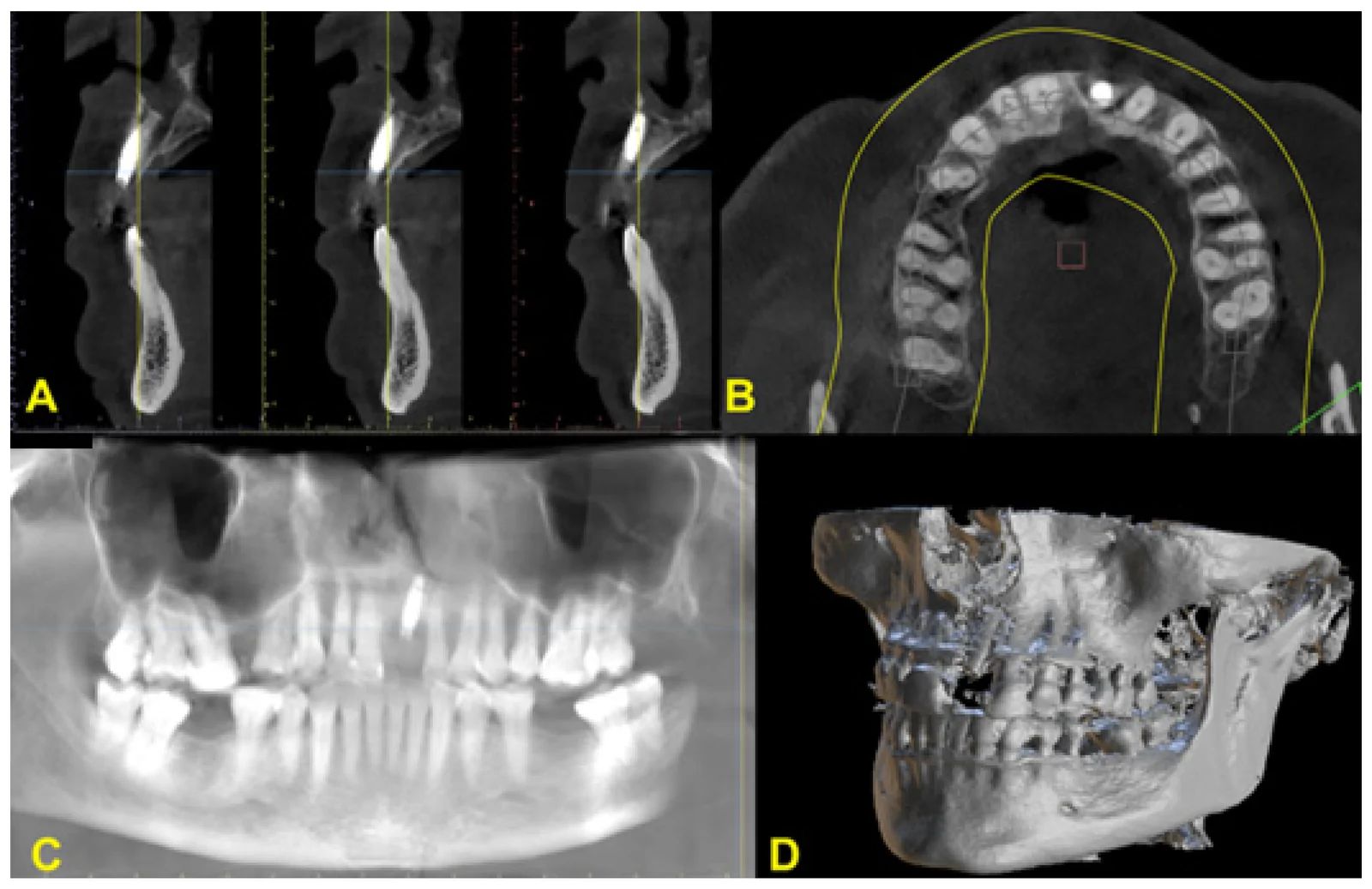
Cone-beam computed tomography of tooth 21 position after first surgery: (**A**) cross-sectional views; (**B**) axial view; (**C**) orthopantomographic view; (**D**) pseudo-3D reconstruction.

Atraumatic extraction of tooth 21 was performed, followed by immediate implant placement. A KISPlant BT implant (4.0 × 10 mm) was inserted into D1-quality bone, achieving a primary stability of 50 Ncm. Intraoral scans were obtained during the same appointment, and a screw-retained PMMA (Poly(methyl methacrylate) synthetic polymer) provisional crown was delivered the following day. The CBCT after first surgery is shown in Figure 8.

#### 2.2.3. Initial Implant and Management

Approximately one month later, the patient returned and reported mobility of the provisional crown and discomfort in the implant region. Clinical examination revealed a fistula in the projection of the implant in the position of tooth 21. The provisional crown was removed, and complete implant destabilization and loss of osseointegration were observed.

During the follow-up interview, the patient admitted non-compliance with the recommended soft diet and excessive loading of the provisional restoration during the healing phase. The overload likely resulted in micromovements exceeding the biological threshold for osseointegration, contributing to inflammatory granulation tissue formation and partial loss of the buccal plate.

The implant was removed atraumatically. Curettage of the inflammatory tissue was performed, and a collagen sponge was inserted to improve hemostasis and support soft-tissue healing. Intraoral scans were obtained for fabrication of a Maryland bridge, which was delivered the following day. The patient was instructed to maintain the provisional restoration for four months to allow for complete healing of the site.

#### 2.2.4. Preoperative Evaluation Prior to Reimplantation

**Figure 9:**
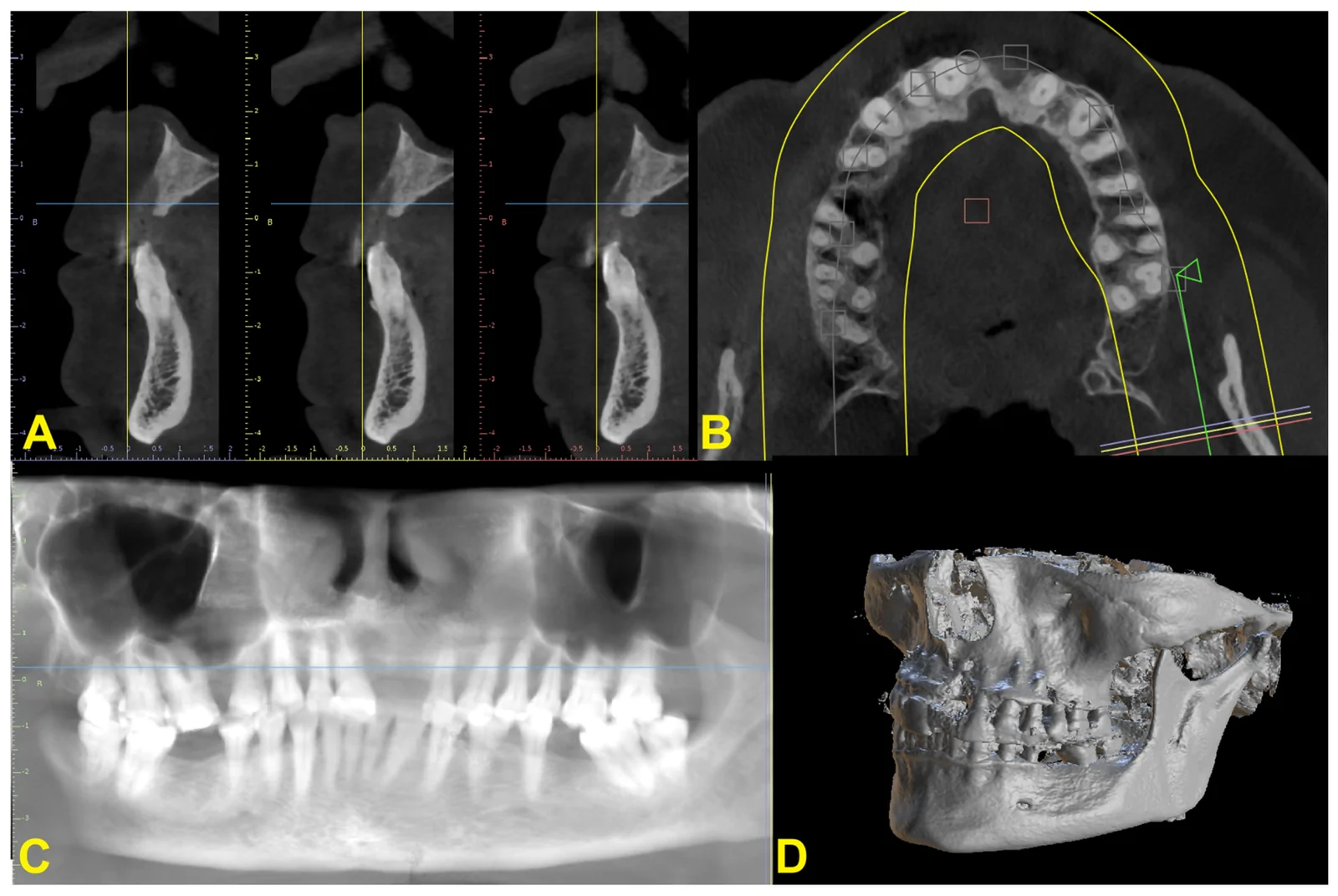
Cone-beam computed tomography of tooth 21 region four months after initial implant procedure: (**A**) cross-sectional views; (**B**) axial view; (**C**) panoramic reconstruction; (**D**) pseudo-3D reconstruction.

**Figure 10:**
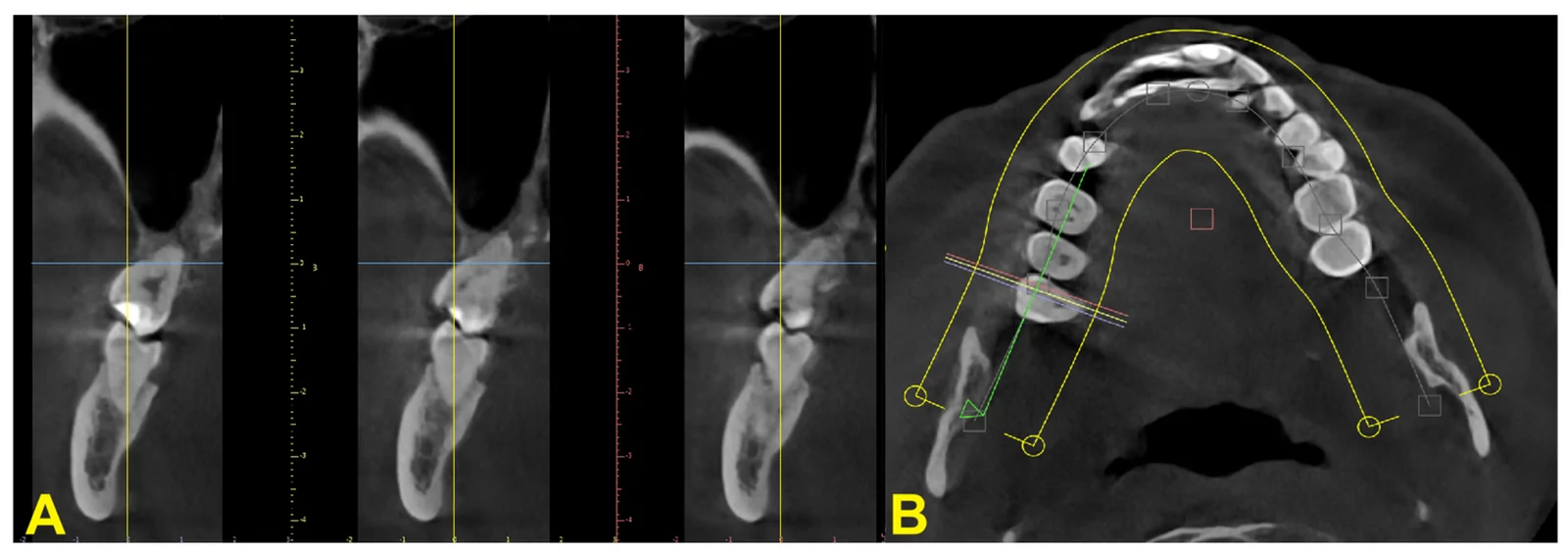
Cone-beam computed tomography of tooth 18 position: (**A**) cross-sectional views; (**B**) axial view.

Three months later, CBCT examination demonstrated severely compromised horizontal ridge dimensions (Figure 9), with the residual ridge width measuring approximately 4 mm. Because of the limited buccal bone volume and previous implant failure, a modified tooth-shell reconstruction was planned using tooth 18 (Figure 10).

#### 2.2.5. Tooth-Shell Reconstruction and Reimplantation

One month later, a second surgical procedure was performed. During this intervention, ridge reconstruction using the tooth-shell technique and simultaneous implant placement were completed within a single surgical procedure. A 3.5 × 11.5 mm KISPlant BT implant (KISPlant, Kuwotech, Republic of Korea) was inserted in a palatal position with bicortical stabilization through engagement of the nasal floor cortical layer to maximize primary stability under compromised anatomical conditions. The implant achieved a primary stability of 35 Ncm. Tooth 18, which was buccally positioned and indicated for extraction, was removed during the same appointment and used to prepare an autologous dentin shell. The tooth was vital but presented superficial carious lesions, which were completely removed before preparation. All soft tissues and pulp were removed, and the enamel was completely stripped, leaving only mineralized dentin.

The dentin fragment was reshaped using a diamond bur and adapted to the buccal contour of the defect. The prepared dentin shell measured approximately 1.5 mm in thickness, 11 mm in height, and 8.5 mm in width, and it was stabilized with a single 1.2 × 6 mm titanium osteosynthesis screw, serving as a rigid buccal wall.

**Figure 11:**
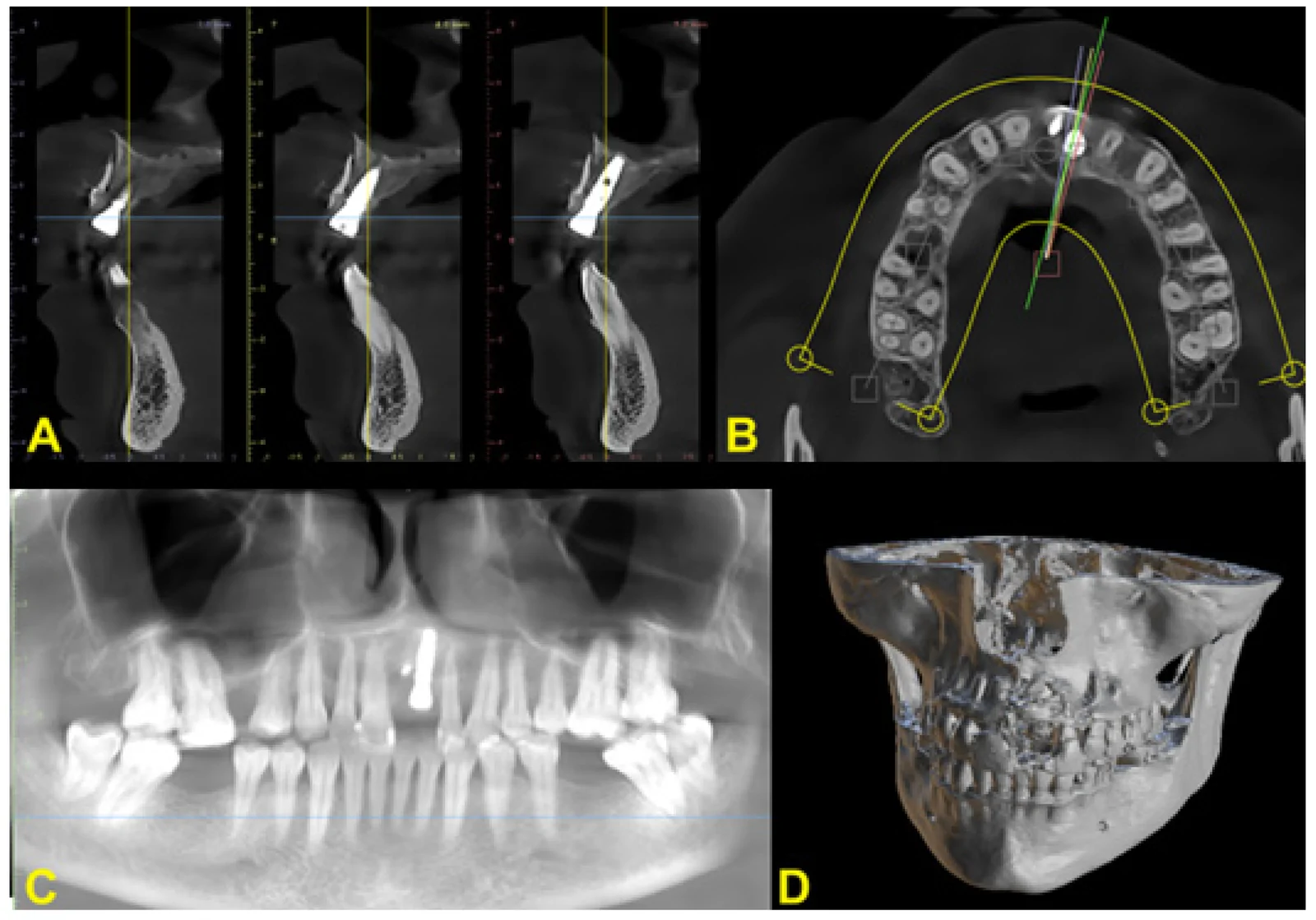
Cone-beam computed tomography of tooth 21 position after second implantation: (**A**) cross-sectional views; (**B**) axial view; (**C**) orthopantomographic view; (**D**) pseudo-3D reconstruction.

Sticky bone was prepared intraoperatively using approximately 0.8–1.0 mL of R.T.R.+ 40/60 biphasic calcium phosphate granules (Septodont, Saint-Maur-des-Fossés, France), composed of 60% hydroxyapatite and 40% tricalcium phosphate, with a particle size of 0.5–1.0 mm. The granules were mixed with autologous advanced platelet-rich fibrin (A-PRF) and injectable platelet-rich fibrin (iPRF). The graft mixture was placed between the dentin shell and residual alveolar ridge. A resorbable bilayer PLGA membrane (R.T.R.+ Membrane, Septodont, Saint-Maur-des-Fossés, France) was placed over the augmented area to support graft containment and soft-tissue healing, and a collagen sponge and PRF membranes were additionally applied to support hemostasis and soft-tissue healing. Primary closure was achieved without tension. A screw-retained PMMA provisional restoration was delivered the following day with a tightening torque of 30 N·cm. Immediate postoperative CBCT imaging confirmed the position of the implant, dentin shell, and augmented ridge (Figure 11).

#### 2.2.6. Follow-Up and Final Prosthetic Rehabilitation

**Figure 12:**
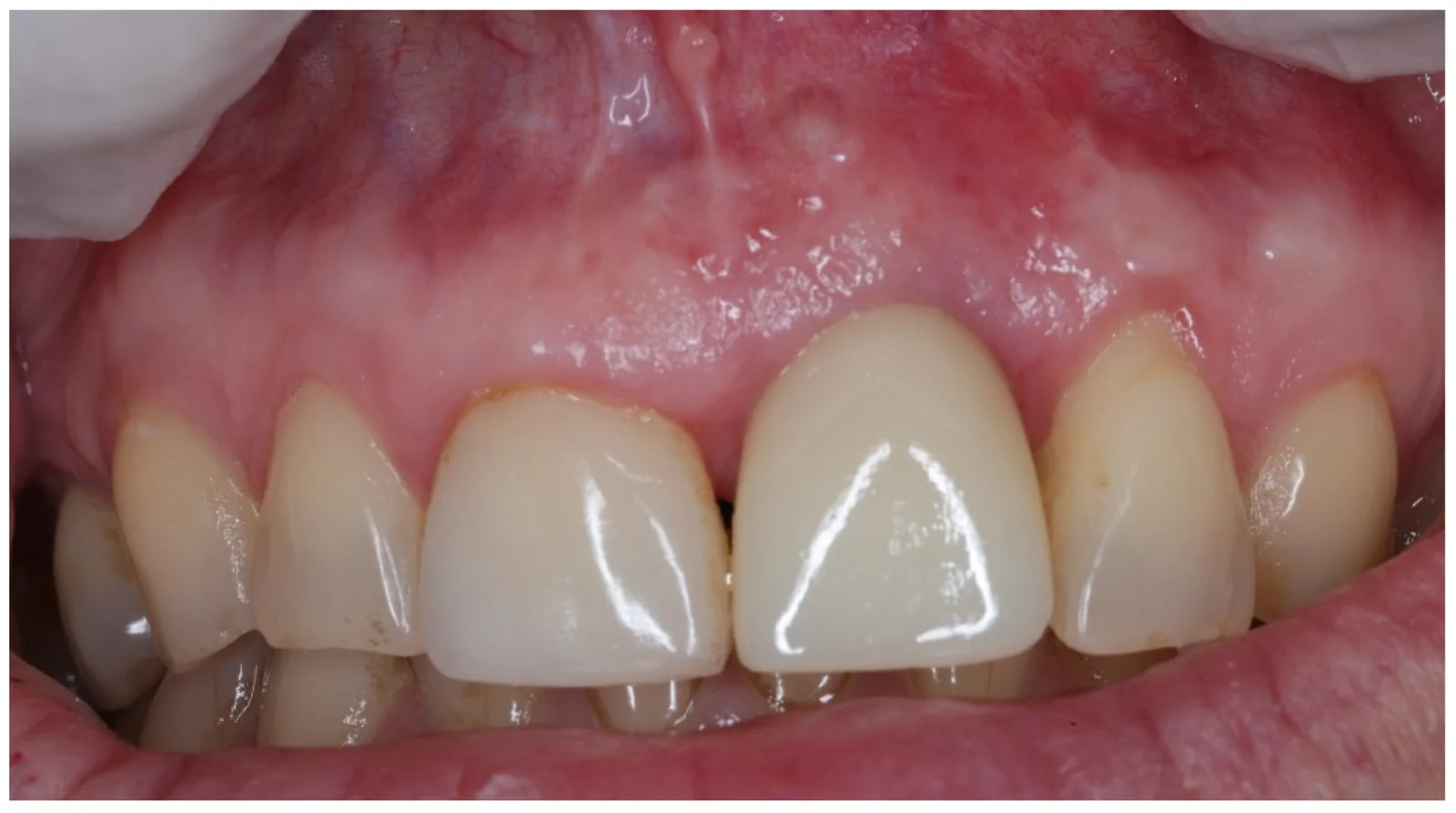
Clinical outcome and definitive prosthetic restoration in Case 2.

**Figure 13:**
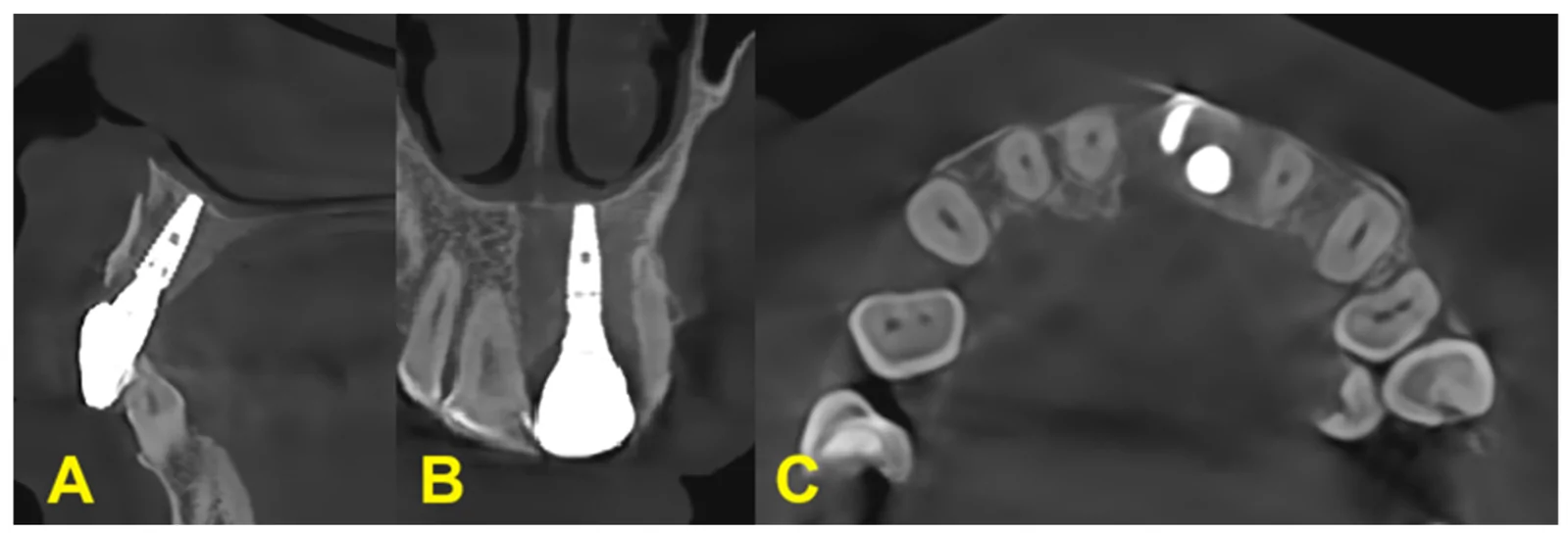
Cone-beam computed tomography of maxillary left incisor position after prosthetic crown delivery: (**A**) cross-sectional views; (**B**) coronal view; (**C**) axial view.

After six months of healing, new intraoral scans were obtained for definitive prosthetic reconstruction. Implant stability quotient (ISQ) measurements exceeded 70, confirming high secondary stability. A screw-retained, full-contour definitive crown with screw channel redirection was subsequently delivered. Clinical and radiographic follow-up demonstrated stable peri-implant conditions, satisfactory maintenance of the reconstructed ridge contour, and no signs of implant mobility, mucosal inflammation, fistula formation, or soft-tissue dehiscence. The osteosynthesis screw was intentionally left in place because it remained asymptomatic and its removal would have required an additional surgical intervention. Complete restoration of the original gingival architecture in the region of tooth 21 was not possible because of the initial extent of buccal hard- and soft-tissue loss. However, no additional soft-tissue augmentation was performed because the patient had a low smile line and considered the final aesthetic outcome satisfactory (Figure 12 and Figure 13).

## 3. Results

In Case 1, the horizontal ridge width increased from approximately 4.1 mm before surgery to approximately 10 mm immediately after augmentation. CBCT examinations performed at 4 and 6 months demonstrated maintenance of the reconstructed ridge contour without clinically relevant dimensional reduction. The implant remained stable and functional. A minor mucosal dehiscence developed around the osteosynthesis screw during healing, resulting in limited scar formation without graft exposure, infection, or the need for additional surgical intervention. Clinical follow-up was continued for 2 years, during which stable hard-tissue dimensions, healthy peri-implant soft tissues, satisfactory aesthetics, and maintenance of implant function were observed.

In Case 2, the residual ridge width before reconstruction was approximately 4 mm and increased to approximately 8 mm after augmentation. The initial immediate implant had previously failed because of stability loss associated with excessive functional loading during the osseointegration period. Following implant removal and complete healing of the site, tooth-shell reconstruction and simultaneous implant placement were successfully performed during a single surgical procedure. At the 6-month follow-up, the implant remained clinically stable, and the ISQ values exceeded 70. No implant mobility, mucosal inflammation, fistula formation, infection, or soft-tissue dehiscence was observed. Complete restoration of the original gingival contour was not achieved owing to the previous loss of buccal hard and soft tissues; however, the aesthetic outcome was considered satisfactory by the patient because of her low smile line.

Throughout the respective observation periods, both dentin shells remained clinically and radiographically stable, and the reconstructed ridge contours were maintained. No graft infection, acute inflammatory reaction, or loss of the implants was observed after definitive prosthetic rehabilitation. Comparative clinical and radiographic findings for both cases are presented in Table 1.

## 4. Discussion

This technical report demonstrates the feasibility of using autologous dentin shells as structural buccal barriers for ridge reconstruction in both primary and salvage implant rehabilitation scenarios. In both presented cases, maintenance of the reconstructed ridge contours and stable clinical and radiographic implant outcomes were observed despite challenging anatomical and clinical conditions.

The biological rationale for the proposed technique is based on the structural and biochemical similarities between dentin and cortical bone. Dentin contains approximately 70% inorganic material, predominantly hydroxyapatite, and an organic matrix composed mainly of type I collagen, bioactive proteins, and growth factors. These molecules participate in mineralized tissue remodeling, osteoblast differentiation, and angiogenesis. Previous experimental and clinical studies have demonstrated that processed autologous dentin may exhibit both osteoconductive and -inductive potential comparable to autogenous bone grafts. The stability of the dentin wall reduces the risk of collapse commonly observed in membrane-based GBR. In contrast to conventional collagen membranes used in guided bone regeneration, dentin shells additionally provide mechanical rigidity and slower osteoclast-mediated resorption, which may contribute to the prolonged maintenance of the graft contour and resistance against soft-tissue collapse during the early healing phase. Furthermore, the ankylotic integration potential of dentin placed in direct contact with bone may facilitate gradual incorporation into surrounding mineralized tissues through replacement remodeling [8,9,10,11,12,13,14,15].

The present technique was inspired by the cortical shell concept introduced by Khoury. However, several important differences should be emphasized. In the classical Khoury technique, cortical plates harvested from the retromolar area require a secondary donor site and additional surgical trauma. In contrast, the proposed autologous tooth-shell technique utilizes extracted teeth as structural barriers, thereby avoiding cortical bone-block harvesting and potentially reducing donor-site morbidity while simplifying the surgical workflow. Although extraction of the donor tooth still represents an additional surgical intervention, its extent is generally less than that associated with harvesting a cortical bone block, particularly when the tooth is already indicated for extraction. Table 2 provides a focused comparison between the modified tooth-shell technique and classical Khoury cortical shell technique [4,6,18,19,20,21,22,23,24,25,26,27,28,29,30].

The first clinical case demonstrated successful ridge augmentation and immediate implant placement in a relatively favorable anatomical situation. However, the second case represented a considerably more demanding salvage reconstruction scenario following overload-related implant failure and buccal plate loss. This distinction is clinically important because it suggests that the proposed technique may also be applicable in selected compromised conditions with reduced bone availability.

The failure of the initial implantation procedure in Case 2 was most likely associated with excessive functional loading during the osseointegration phase. The patient admitted non-compliance with postoperative dietary recommendations, which probably resulted in excessive micromovements exceeding the biological threshold required for stable osseointegration. This observation highlights the critical importance of postoperative compliance in immediate loading protocols, particularly in cases involving limited residual bone volume and simultaneous augmentation procedures. Despite the previous implant failure, secondary reconstruction using an autologous dentin shell enabled successful reimplantation with stable peri-implant conditions after 6 months. Achieving adequate primary stability was essential for immediate provisionalization in both cases. Implant positioning was carefully optimized to maximize cortical engagement and prosthetically driven alignment. Bicortical stabilization through engagement of the nasal floor cortical layer was required in Case 2 because of the limited residual bone volume.

An additional advantage of immediate provisionalization was the ability to guide peri-implant soft-tissue healing and emergence profile formation during the maturation phase. Nevertheless, the aesthetic outcome remained partially limited in Case 2 because of the previous loss of buccal hard and soft tissues associated with implant failure and inflammatory destruction. Although complete restoration of the original gingival architecture could not be achieved, the patient considered the final aesthetic outcome satisfactory because of her low smile line.

The proposed technique may be particularly useful in selected cases involving a thin or deficient buccal plate, simultaneous implant placement, previous implant failure, or a need for ridge reconstruction without cortical bone-block harvesting. However, the availability of a suitable donor tooth remains an important prerequisite. In the presented cases, the donor teeth were already indicated for extraction, thereby limiting the additional biological cost of the procedure.

The use of biphasic calcium phosphate instead of particulate dentin was related primarily to the limited amount of residual dentin available after preparation of the structural shell. Most of the usable tooth tissue was preserved to create a rigid buccal barrier, while R.T.R.+ 40/60 provided a standardized and sufficient particulate volume for filling the defect. The use of R.T.R.+ 40/60, comprising hydroxyapatite and tricalcium phosphate, was intended to maintain initial space with gradual remodeling. In this modified approach, the dentin was therefore used primarily as a structural autologous barrier rather than as the sole particulate grafting material.

Several contraindications should also be considered, including acute purulent infection, uncontrolled periodontal disease, severe parafunctional habits, poor patient compliance, and insufficient operator experience. Potential complications include incomplete incorporation or infection of the dentin shell, screw exposure, and mucosal dehiscence. A minor mucosal dehiscence occurred in Case 1; however, it was not associated with graft exposure, infection, or the need for an additional surgical intervention. Long-term biological behavior and dimensional stability remain to be established in larger clinical studies. Nevertheless, the clinical and radiographic findings observed during the available follow-up periods support the feasibility of the technique in carefully selected cases [18,19,20,21,22,23,24,25,26,27,28,29,30].

The modified tooth-shell technique should be regarded as highly operator-dependent and surgically demanding. Successful execution requires advanced knowledge of implant positioning, ridge augmentation, soft-tissue management, and immediate provisionalization protocols. Precise dentin shell preparation, stable fixation, tension-free primary closure, adequate implant stability, and strict control of occlusal loading are important factors influencing successful healing.

Recent studies investigating autologous dentin grafts have reported favorable dimensional stability and implant survival outcomes. The findings of the present technical report are consistent with previous reports by Pang et al. and Gualini et al. supporting the promise of tooth-derived biomaterials for regenerative implant procedures [24,25]. However, direct comparison with established augmentation methods remains limited because of differences in clinical indications, processing protocols, outcome measures, and available follow-up periods.

### Study Limitations

The present report has several limitations. Only two clinical cases were included, and the observation periods were unequal, with 2 years of clinical follow-up in Case 1 and 6 months in Case 2. No control group was available, and the two cases differed in their initial clinical conditions and treatment histories. Histological assessment was not performed because obtaining a hard-tissue biopsy from clinically stable and successfully treated sites would have required an additional invasive procedure without a therapeutic indication. Consequently, the biological nature and degree of remodeling of the tissues surrounding the dentin shells could not be directly confirmed.

In addition, ridge changes were assessed using selected CBCT measurements rather than a standardized three-dimensional volumetric analysis. The reported measurements therefore document clinically relevant dimensional changes at the treated sites but should not be interpreted as a complete quantitative assessment of the regenerated tissue volume. Complete intraoperative photographic documentation was also unavailable. To improve reproducibility, the surgical description was expanded to include the approximate dimensions of the dentin shells, fixation screws, graft materials, membranes, and treated areas. Furthermore, no standardized protocol currently exists regarding the optimal dentin shell thickness, preparation method, or long-term remodeling dynamics. The procedure also remains strongly dependent on operator experience, which limits the generalizability of the presented findings.

Future developments may include the digitally planned or CAD/CAM-assisted preparation of dentin shells, including the use of three-dimensional printed analogs or templates to facilitate preoperative planning, improve shell adaptation, and potentially reduce surgical time. Further prospective studies involving larger patient groups, standardized radiographic measurements, longer follow-up periods, and histological evaluation when clinically and ethically justified are required to assess the biological behavior, dimensional stability, and predictability of autologous dentin shells used as structural barriers in implant dentistry.

Based on the preliminary clinical observations presented in this technical report and the current biological understanding of autologous dentin grafting, the authors propose potential indications and contraindications for the tooth-shell technique in implant reconstruction procedures. These recommendations should be regarded as hypothesis-generating rather than as definitive clinical guidelines. Careful patient selection, sufficient primary implant stability, appropriate donor-tooth availability, and strict postoperative compliance appear particularly important when the technique is combined with immediate provisionalization (Table 3).

Despite the promising clinical and radiographic outcomes observed in the present report, the proposed tooth-shell technique should be considered technically demanding and dependent on advanced surgical and implantological experience. Successful execution requires precise ridge augmentation planning, accurate dentin shell preparation, appropriate implant positioning, and careful soft-tissue management. In both presented cases, intraoperative decision making was necessary to optimize the implant trajectory, cortical engagement, and prosthetically driven positioning within the aesthetic zone.

Particular attention should be paid to achieving tension-free primary closure over the augmented area. Adequate flap release, periosteal mobilization, and careful manipulation of the mucogingival tissues are essential to protect the graft and reduce the risk of wound dehiscence. Immediate provisionalization further increases procedural complexity and requires careful prosthetic planning, sufficient primary implant stability, and strict control of occlusal loading during the osseointegration period.

Case 2 also emphasizes the importance of patient compliance. Excessive functional loading of the provisional restoration during early healing likely contributed to implant destabilization and loss of osseointegration following the initial implantation procedure. Therefore, the proposed technique should be reserved for carefully selected and cooperative patients and performed by clinicians experienced in implant placement, ridge augmentation, soft-tissue management, and immediate provisionalization.

Although fully guided implant surgery is increasingly used in contemporary implantology, its applicability may be limited in complex reconstructive procedures requiring simultaneous ridge augmentation and dynamic soft-tissue management. Static guided workflows may reduce the surgeon’s ability to modify implant positioning in response to the actual residual bone volume, bone density, dentin shell position, and intraoperative anatomical conditions.

In the presented cases, implant osteotomy preparation required continuous adaptation to achieve appropriate prosthetically driven alignment and cortical engagement. In Case 2, bicortical stabilization through engagement of the nasal floor cortical layer required precise intraoperative control. Such modifications may be difficult to perform using a fully guided static template.

Furthermore, flap mobilization, periosteal release, dentin shell adaptation, and tension-free wound closure require direct surgical access and cannot be controlled through a static implant guide alone. The authors believe that fully guided implant surgery may not be optimal in highly complex reconstructive procedures requiring simultaneous hard- and soft-tissue augmentation. Digital planning may nevertheless remain valuable for preoperative anatomical assessment, implant trajectory planning, and prosthetic design.

## 5. Conclusions

The autologous tooth-shell technique appears to be a promising biologically based approach for horizontal ridge augmentation performed simultaneously with implant placement and provisionalization. The use of processed autologous dentin as a buccal shell may help maintain the reconstructed ridge contour, avoid cortical bone-block harvesting, and potentially reduce donor-site morbidity while providing satisfactory short-term clinical and radiographic outcomes in appropriately selected patients.

The presented cases provide preliminary observations suggesting that this approach may be applicable both in primary implant therapy and in more demanding reconstruction procedures following implant failure and ridge deficiency. An additional potential advantage of the technique is the combination of hard-tissue augmentation and prosthetically driven implant rehabilitation within a single surgical procedure.

At the same time, the procedure remains technically demanding and requires experience in implant surgery, ridge augmentation, and peri-implant soft-tissue management. Proper case selection, sufficient primary implant stability, careful control of occlusal loading, and good patient compliance during healing are important factors that may influence treatment outcomes.

The findings of this two-case technical report should be considered hypothesis-generating rather than definitive evidence of clinical predictability or superiority. Further prospective studies involving larger patient groups, standardized dimensional assessment, and longer observation periods are required to evaluate the long-term biological behavior and stability of autologous dentin shells used for implant-site reconstruction. Histological evaluation may provide additional information when clinically and ethically justified.

## Figures and Tables

**Table 1:** Summary of clinical and radiographic data for two presented cases.

Parameter	Case 1	Case 2
Age	29	58
Sex	Female	Female
Systemic Condition	Endometriosis	Hypertension
Implant Region (FDI)	23	21
Source Tooth	Impacted 28 (buccally positioned)	Extracted 18 (buccally positioned)
Ridge Width Before Augmentation	4.1 mm	4.0 mm
Ridge Width After Augmentation	10 mm	8 mm
Dentin Shell Dimensions	2.0 × 8.0 × 9.0 mm	1.5 × 11.0 × 8.5 mm
Implant Timing	Standard implantation	Delayed reimplantation
Implant Dimensions	4.0 × 10 mm	3.5 × 11.5 mm
Primary Stability	45 Ncm	35 Ncm
Immediate Provisionalization	Yes	Yes
Complications	Minor mucosal dehiscence without graft exposure or infection	None after tooth-shell reconstruction
ISQ At Follow-Up	Not measured	>70
Total Clinical Follow-Up	2 years	6 months
Radiographic Follow-Up	4 and 6 months	6 months
Definitive Prosthetic Restoration	Zirconia-based screw-retained crown	Zirconia-based screw-retained crown with screw channel redirection
Final Outcome	Stable	Stable

**Table 2:** Focused comparison between modified tooth-shell technique and classical Khoury cortical shell technique.

Feature	Classical Khoury Cortical Shell Technique	Modified Tooth-Shell Technique
**Main Graft Material**	Autogenous cortical bone	Autologous dentin shell combined with biphasic calcium phosphate and PRF
**Structural Stability**	High	High because of rigid dentin shell
**Space-Maintaining Capacity**	High	High
**Need For Donor Site**	Yes, cortical bone harvesting from mandibular ramus or retromolar region required	Yes, extracted tooth required
**Donor-Site Morbidity**	Moderate to high	Minor when tooth is already indicated for extraction
**Need For Barrier Membrane**	Usually not essential because of rigid cortical shell	Used as adjunct for graft containment
**Expected Remodeling And Resorption**	Variable; dependent on graft thickness and vascularization	Expected gradual remodeling while maintaining initial structural support
**Biological Characteristics**	Osteogenic, osteoinductive, and osteoconductive	Structural autologous dentin barrier combined with osteoconductive particulate graft and PRF
**Surgical Complexity**	High	High and operator-dependent
**Treatment Stages**	Usually one augmentation procedure, with implant placement either simultaneous or delayed	Simultaneous ridge reconstruction and implant placement in presented cases
**Main Advantages**	Excellent mechanical stability and autogenous biological potential	Avoids cortical bone-block harvesting and provides rigid autologous structural barrier
**Main Limitations**	Donor-site morbidity, surgical time, and technical difficulty	Requires suitable tooth and advanced surgical skills and currently has limited clinical evidence
**Current Level Of Evidence**	Moderate to high	Low; currently based mainly on technical reports and small clinical series

**Table 3:** Preliminary considerations regarding potential indications and contraindications for the autologous tooth-shell technique.

Clinical Condition	Preliminary Recommendation
Thin or deficient buccal plate	Potential indication
Simultaneous implant placement	May be considered in selected cases
Previous implant failure	May be considered in carefully selected salvage cases
Horizontal ridge deficiency	Potential indication
Simultaneous immediate provisionalization	May be considered in experienced hands
Severe vertical ridge defects	Limited indication
Acute purulent infection	Contraindication
Active untreated periodontitis	Relative contraindication
Heavy smoking	Relative contraindication
Severe parafunctional habits	Relative contraindication
High smile line with severe soft-tissue loss	Use with caution
Poor patient compliance	Contraindication
Inexperienced operator	Contraindication
Infected donor tooth	Relative contraindication
Absence of suitable donor tooth	Technique not applicable
Insufficient primary implant stability	Immediate provisionalization not recommended

## Data Availability

The data presented in this study are available from the corresponding author upon request. The data are not publicly available due to privacy restrictions.

## Abbreviations

ASA	American Society of Anesthesiologists scale
BMPs	Bone morphogenetic proteins
CBCT	Cone-beam computed tomography
DMP-1	Dentin matrix protein-1
DSPP	Dentin sialophosphoprotein
FDI	World Dental Federation notation
GBR	Guided bone regeneration
iPRF	Injective platelet-rich fibrin
ISQ	Implant stability quotient
PMMA	Poly(methyl methacrylate) synthetic polymer
PRF	Platelet-rich fibrin
TGF-β	Transforming growth factor beta
